# Changes in the Gut Microbiota of Patients After SARS-CoV-2 Infection: What Do We Know?

**DOI:** 10.3390/microorganisms13112529

**Published:** 2025-11-04

**Authors:** Isabel de Souza Andrade Arruda, Caio da Silva Cavalcante, Rebeca Siqueira Rubens, Larissa Nava Pinto de Faria Castro, Yanna Karla de Medeiros Nóbrega, Tanise Vendruscolo Dalmolin

**Affiliations:** Laboratório de Microbiologia e Imunologia Clínica (LabMIC), Departamento de Farmácia, Faculdade de Ciências da Saúde, Universidade de Brasília (UnB), Brasília 70910-900, Brazil; isabelarruda21@gmail.com (I.d.S.A.A.); caioscfarma@gmail.com (C.d.S.C.); rebecarubens9@gmail.com (R.S.R.); larissa.navajob@gmail.com (L.N.P.d.F.C.); yannanobrega@gmail.com (Y.K.d.M.N.)

**Keywords:** dysbiosis, long-COVID-19, gut microbiota, post-acute COVID-19 syndrome, SARS-CoV-2, gut–lung axis, brain–gut axis

## Abstract

COVID-19 can cause long-term symptoms, such as a post-infection syndrome, known as Long-COVID. Among the symptoms present during this period, the most reported are gastrointestinal symptoms. This study discusses the effects of changes in the gut microbiota of post-COVID-19 patients. SARS-CoV-2 infection is associated with significant alterations in gut microbial composition, disturbing its homeostasis and promoting a reduction in the abundance of beneficial symbiotic bacteria and an increase in the abundance of opportunistic pathogens. Furthermore, the composition of the gut microbiota may play a role in the prognosis of patients with post-COVID-19 infection. The microbiota of the intestinal tract and the respiratory tract influence each other; therefore, the gut–lung axis has attracted increasing interest in understanding COVID-19. Moreover, the brain–gut axis has been studied, since there have been reports of anxiety and depression along with post-COVID-19 gastrointestinal symptoms. Treatments options for intestinal dysbiosis in Long-COVID patients include probiotics, prebiotics, and fecal microbiota transplantation. These treatments may serve as an approach to improve gastrointestinal symptoms during Long-COVID, increasing microbiome diversity, strengthening the integrity of intestinal barrier functions, and consequently influencing the treatment of COVID-19.

## 1. Introduction

On 30 January 2020, the World Health Organization (WHO) declared that the pandemic of the new coronavirus SARS-CoV-2, which causes the disease COVID-19, was considered a public health emergency of international importance [1]. Currently, COVID-19 has a prevalence of more than 775 million confirmed cases, resulting in more than 7 million deaths worldwide [2].

SARS-CoV-2 is a single-stranded RNA virus with a high rate of evolution and is highly contagious. Several variants have already been identified, each with a higher infectivity rate. Therefore, a series of vaccines has been developed to contain the virus [3].

The main symptoms of COVID-19 are fever, headache, dry cough, sore throat, shortness of breath, fatigue, and the loss of smell and taste [3,4,5]. Among the symptoms of COVID-19 are also gastrointestinal problems, the most reported being diarrhea, vomiting, the loss of appetite, abdominal pain, constipation, nausea, and dysphagia [3,5,6,7,8,9].

COVID-19 can also cause long-term symptoms, such as a post-infection syndrome, known as Long-COVID. The WHO defined this condition as symptoms that occur three months after the onset of SARS-CoV-2 infection and may last for at least two months or more [3]. Among the symptoms present during this period, the most reported and researched are gastrointestinal symptoms [10].

The prevalence of Long-COVID varies widely and should take into account previous symptoms. A cohort study compared a group of people infected with laboratory-confirmed SARS-CoV-2 with another group who had never been infected (group of healthy controls) and found that the true prevalence of Long-COVID was 6.6%, 6.5%, and 10.4% at 6, 12, and 18 months, respectively, in Scotland [11].

The gastrointestinal tract, together with its diverse microbial population, is the largest immune organ in the body, with the microbiota responsible for modulating immune responses [12]. The gut microbiota is composed of a community of microorganisms, including bacteria, archaea, fungi, viruses, and protozoa. Gut bacteria comprise six major bacterial phyla: Bacillota (former phylum Firmicutes—*Enterococcus*, *Lactobacillus*, *Veilonella*, *Ruminococcus*, *Roseburia*, *Clostridium*, among others), Bacteroidota (former phylum Bacteroidetes—*Prevotella*, *Bacteroides*, among others), Pseudomonadota (former phylum Proteobacteria), Actinomycetota (former phylum Actinomycetes—*Bifidobacterium*, *Collinsella*), Verrucomicrobiota (former phylum Verrucomicrobia), and Fusobacteriota (former phylum Fusobacteria) [13,14].

A complex community of microorganisms in the gut microbiota can induce and develop the host’s immune system, allowing it to combat opportunistic pathogens and promote an adequate response [15]. Furthermore, the microbiota in homeostasis produces short-chain fatty acids (SCFAs) such as acetate, propionate, and butyrate. SCFAs are recognized for their immunomodulatory and anti-inflammatory functions in the gut (maintaining proper intestinal barrier) and other distal mucosal sites. SCFA levels may be reduced during the period of COVID-19 infection, which is correlated with greater severity of the disease [16,17,18,19,20].

The microbiota of an unhealthy individual differs significantly from the microbiota of a healthy one, which influences systemic inflammation in patients with COVID-19. Microbiota dysbiosis can lead to an abnormal production of inflammatory cytokines and a hyperstimulation of T cells, both changes being relevant in COVID-19 [21,22,23].

Therefore, there is a very strong relationship between the gut microbiota and COVID-19, with the microbiota playing a fundamental role in the host’s immune response to respiratory viral infection. The disturbance in homeostasis leads to dysbiosis in the host, affecting a possible response against viral infection [24,25,26].

Dysbiosis is characterized by a change in the gut microbiota that is involved in homeostasis, which can cause the proliferation of microorganisms that are harmful to the host’s health. These harmful bacteria can cause increased production of certain endotoxins, which are subsequently released into the bloodstream, causing a severe immune system response, producing an increase in inflammation resulting from the release of pro-inflammatory cytokines [17].

To date, it is known that SARS-CoV-2 infection is associated with significant alterations in gut microbial composition, disturbing its homeostasis and promoting a reduction in the abundance of beneficial symbiotic bacteria and an increase in the abundance of opportunistic pathogens [3]. Despite the existence of studies on the composition and changes in the gut microbiota during infection, little is known about why these symptoms and changes persist after the recovery period [10,27]. Furthermore, post-COVID dysbiosis has often been associated with the severity of the disease [3]. Therefore, this study discusses the effects of changes in the gut microbiota in post-COVID-19 patients.

## 2. Gut–Lung and Gut–Brain Axis

The gut–lung axis has attracted increasing interest, as there is evidence that the microbiota of the intestinal tract and the respiratory tract influence each other. Angiotensin-converting enzyme 2 (ACE-2) is one of the main primary receptors for the entry of the SARS-CoV-2 virus into human cells, being highly expressed in the respiratory and intestinal tracts, among other organs. During SARS-CoV-2 infection, the ACE-2 enzyme may decrease its expression to aid viral replication and promote infection. This can cause the accumulation of angiotensin-2 and subsequently a disturbance in the homeostasis of the renin–angiotensin system, impacting the malabsorption of amino acids, intestinal inflammation, and the dysfunction of the intestinal barrier, which is beneficial for the entry of opportunistic pathogens [15,28,29,30,31,32].

ACE-2 is highly expressed in the mucosa of the small intestine, which may be one of the biological mechanisms associated with the gastrointestinal symptoms of COVID-19, facilitating the translocation of foreign molecules, including the virus itself, into the blood circulation, triggering systemic inflammatory responses that compromise multiple organs. During SARS-CoV-2 infection, the lung microbiota is also affected, with a possible depletion of alveolar macrophages that contribute to the complications and worsening of COVID-19 [15,33].

**Figure 1:**
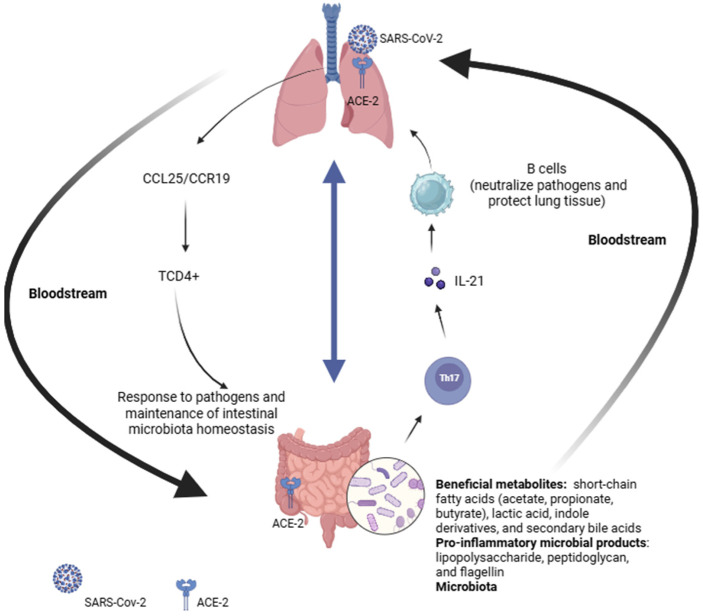
Overview of gut–lung axis. ACE-2: Angiotensin-converting enzyme 2; CCL25: chemokine (C-C motif) ligand 25; CCR19: C-C chemokine receptor type 19; IL-21: interleukin 21; TCD4^+^: CD4^+^ T helper cells; Th17: T helper 17 cells. Created in BioRender.com.

Some interactions of the immune system may explain the functionality of the gut–lung axis. The segmented filamentous bacteria of the intestine, which are generally part of the commensal microbiota, can activate the Th17 response (a subpopulation of CD4^+^ T Lymphocytes), which plays an important role in modulating the immune system, including the activation of memory B Lymphocytes in the lungs. The cytokines produced by Th17 cells, especially IL-21, promote the activation and differentiation of B Lymphocytes into plasma cells, which are responsible for the production of antibodies. These antibodies are essential for neutralizing pathogens and protecting the lung tissue [34]. Conversely, the CCL25/CCR9 pathway induces the recruitment of lung-derived CD4^+^ T lymphocytes to the intestinal tract, which is relevant both for the response to pathogens and for the maintenance of gut microbiota homeostasis. The change in the migration of CD4^+^ T Lymphocytes due to the CCL25/CCR9 pathway can modify the intestinal environment, affecting the microbiota [3] (Figure 1).

The gut–lung axis is a bidirectional system that works like a tube, in which endotoxins and metabolites produced by intestinal microorganisms reach the lungs through the bloodstream, causing inflammation. Evidence suggests that this axis influences the progression of inflammatory cytokines during COVID-19, resulting in a “cytokine storm” that can cause significant damage to the body [33]. This dysfunction can lead to immune disorders and gastrointestinal symptoms. Additionally, the prolonged presence of fecal viral RNA in post-COVID-19 patients suggests a slower recovery of the gut microbiome compared to the oral one [35].

The severity of gastrointestinal symptoms may be an indicator of the severity of the infection, suggesting that the presence of gastrointestinal symptoms may present a prognosis for the development of severe neurological symptoms, strengthening the idea of the gut–brain axis as well. Evidence shows that SARS-CoV-2 RNA was detected both in the central nervous system and in the feces of patients who were positive for the infection, and the microbiota of these patients revealed bacterial pathogens from the respiratory tract, suggesting a possible interrelationship between the gut–lung axis and gut–brain axis [36].

The gut–brain axis constitutes an interconnected system involving neuropeptides, inflammatory markers, microbiota, and hormones [36]. The communications channels within this complex network have not yet been fully elucidated. However, it is known that the gut microbiota modulates the immune system and, subsequently, the nervous system through the stimulation of local and systemic immune responses [36]. Furthermore, the gut microbiota influences central nervous system function through the synthesis of neurotransmitters and mediators such as 5-hydroxytryptamine, histamine, melatonin, acetylcholine, and catecholamines [36].

Some patients have reported symptoms such as anxiety and depression following the acute period of SARS-CoV-2 infection, and some of these symptoms have been reported alongside gastrointestinal symptoms [36]. The psychosocial stress caused by the pandemic has caused an increase in intestinal permeability, resulting in gastrointestinal symptoms, such as abdominal pain and an abnormal influx of food antigens and bacteria, thus disturbing systemic immune homeostasis and compromising brain structure and function. Psychological factors can also delay gastric emptying, leading to dysplasia and increasing the risk of dyspepsia and intestinal inflammation [37,38].

**Figure 2:**
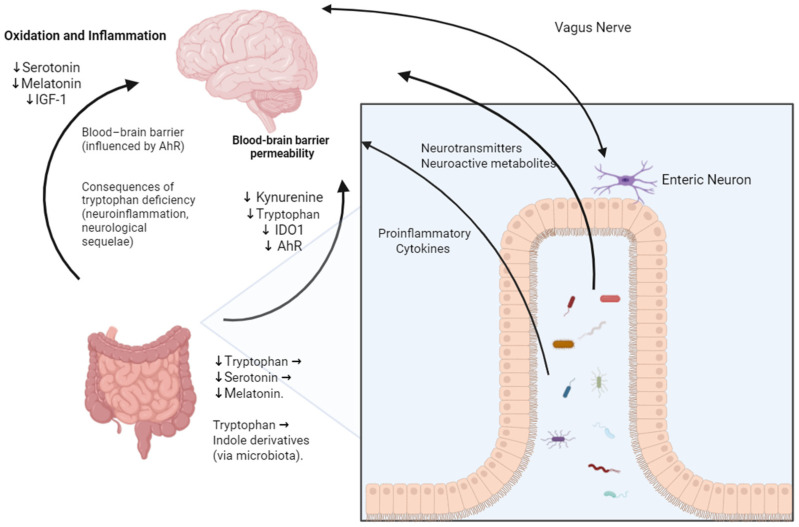
Overview of the gut–brain axis. AhR: aryl hydrocarbon receptor; IDO1: indoleamine 2,3-dioxygenase 1; IGF-1: insulin-like growth factor 1. ↓ Increase; ↑ Decrease; → Sequence, Direction, Consequence. Created in BioRender.com.

Patients with COVID-19 showed lower levels of tryptophan, correlated with the severity of the infection, affecting the production of kynurenine (metabolite of the amino acid tryptophan) and the activity of the enzyme IDO1 (present especially in immune cells such as macrophages that catalyze the degradation of tryptophan into kynurenine), impacting inflammatory responses during illness [36]. A low expression of the IDO1 enzyme decreases the activity of the aryl hydrocarbon receptor (AhR), which is a cytosolic nuclear receptor and transcription factor that, when activated by ligands, such as kynurenine metabolites, translocates to the cell nucleus and regulates the expression of genes involved in the immune response, in the homeostasis of mucosal barriers, and in the detoxification of xenobiotic compounds, thus being crucial for maintaining mucosal barriers such as the blood–brain barrier, and explaining the neurological sequelae of COVID-19. Low levels of tryptophan also affect the production of serotonin and melatonin, which have antioxidant and anti-inflammatory effects, and insulin-like growth factor type 1 (IGF-1), which is a hormone with important functions in cell growth and development and plays a critical role in viral inflammation and acute respiratory distress syndrome [39] (Figure 2).

Decreased microbial tryptophan biosynthesis is observed in patients with more severe increases in post-COVID-19 gastrointestinal symptoms, as well as being correlated with an increased severity of mental health symptoms. As a result, some patients who reported sadness six months after the initial diagnosis of COVID-19 had a lower concentration of serotonin, demonstrating that there is possibly a link between patients with COVID-19 and long-term disorders resulting from gut–brain interactions [40,41].

However, it is worth noting that lower tryptophan concentrations were observed in patients who only presented symptoms of anxiety/depression related to COVID-19 but were without gastrointestinal symptoms. Therefore, it is not clear whether these symptoms related to mental health are due to the virus, the consequences of hospitalization, other symptoms, or stress factors related to the pandemic [40]. It is therefore necessary to develop more studies and correlate these factors, so that it is possible to understand the relationship between them.

In terms of treatment, the enzyme transmembrane serine protease 2 (TMPRSS2), present in the intestinal epithelium, is crucial for the activation and entry of the SARS-CoV-2 virus into host cells. The protein facilitates the entry of the virus into cells by cleaving the viral spike protein, activating it for fusion with the cell membrane, and exposing the fusion peptide necessary for viral entry. The inhibition of the TMPRSS2 enzyme can block viral entry into the intestine and blood–brain barrier, reducing the severity of the infection. In parallel, zonulin is a protein that regulates the permeability of intercellular junctions in the intestine and the blood–brain barrier. Increases in zonulin levels can lead to greater permeability, facilitating the passage of viral particles into the bloodstream. Larazotide acetate (AT1001), a synthetic zonulin inhibitor, can reduce the entry of viral particles into the bloodstream, decreasing the severity of the infection [36].

## 3. Change in the Bacterial Population of the Gut Microbiota After SARS-CoV-2 Infection

The gut microbiota is made up of more than 10^4^ microorganisms of different species that encode 200 times the number of human genes [13]. Changes in the gut microbiota have been reported in patients diagnosed with COVID-19, mainly after the period of infection (Long-COVID).

One of the most reported changes is the change in its composition, with an increase in opportunistic pathogens and a decrease in beneficial commensal bacteria observed when compared to healthy individuals [5,9].

Studies carried out with fecal samples from patients in the acute phase of infection and after the period of SARS-CoV-2 infection reported that the composition of the gut microbiota is totally related to the presence of the virus and the severity of the infection, as it remains vulnerable and suffers disturbances [42]. These disorders may also be caused by the increased use of antimicrobials, with patients showing a large decrease in bacteria beneficial to the gut microbiota [3,9,37].

Patients diagnosed with COVID-19 presented with many opportunistic bacteria that are correlated with the presence of infection, *Acinetobacter*, *Pseudomonas*, *Actinomyces viscosus*, and *Enterococcus*, and those of the order Enterobacterales, such as *Citrobacter* [3,27,43,44]. Common opportunistic pathogens, such as *Enterococcus* and species from the order Enterobacterales, are found in abundance, especially in critically ill patients [4,45].

In patients with post-acute COVID-19 or Long-COVID syndrome, the species *Bacteroides vulgatus* and *Ruminococcus gnavus* were in greater abundance [5,46]. Alterations in the gut microbiota led to immune dysregulation, which can subsequently result in a cytokine storm during infection. Consequently, this cytokine storm may be positively associated with an increase in *Bacterioides* spp. [27]. After one and three months of infection, a decrease was reported when compared to acute samples of COVID-19 [45]. Furthermore, *Oscillibacter* and *Parabacteroides* were increased in patients who tested positive for SARS-CoV-2 [5,45].

In analyses of fecal samples from patients with a higher degree of infectivity, the abundant presence of *Streptococcus infantis* and *Morganella morganii* was observed [4]. *S. infantis* is normally found in the upper respiratory tract and oral cavity, suggesting the occurrence of a passage of extra-intestinal bacteria to the intestine in the context of COVID-19. *M. morganii*, belonging to the order Enterobacterales, is a bacterium commonly found in the intestinal tract and is associated with opportunistic human infections [47].

Patients who had severe COVID presented many opportunistic species belonging to the Bacillota phylum, such as *Clostridium ramosum*, *Coprobacillus*, and *Clostridium hathewayi*, which are correlated with the severity of the disease [4,5,35,37]. Furthermore, the phyla Pseudomonadota also showed an increase in their quantity [3,27,43,44]. Therefore, patients with a more severe form of infection had an increase in opportunistic pathogens in their gut microbiota [9].

On the other hand, patients with COVID-19 presented a decrease in beneficial and symbiotic bacteria, important for regulating the gut microbiota: *Lachnoclostridium*, *Eggerthella*, *Anaerostipes*, the Lachnospiraceae family, *Enterocloster*, and *Flavonifractor*. Furthermore, a decrease was observed in bacteria producing short-chain fatty acids such as *Bifidobacterium*, *Faecalibacterium*, and *Roseburia*. These microorganisms are very important for intestinal health in terms of reducing inflammation and regulating the immune system [3,43,44]. The abundance of beneficial bacteria, such as *Faecalibacterium* spp., *Fusicatenibacter saccharivorans*, *Roseburia hominis*, *Alistipes onderdonkii*, *Eubacterium rectale*, and *Bifidobacterium*, was inversely related to disease severity [4,5,35,37,48,49].

Anti-inflammatory bacteria, which would be beneficial and help in the treatment of the infection, such as *Faecalibacterium prausnitzii*, were found in smaller quantities and were inversely related to the severity of the disease [9,35], while the amount of *Alistipes* was observed to increase during recovery from the disease [45].

*Eubacterium rectale*, *Roseburia*, and, to a lesser extent, *Faecalibacterium prausnitzii* are butyrate-producing bacteria and their presence was observed to increase in fecal samples from infected patients three months after infection compared to acute COVID-19 samples. Butyrate maintains the integrity of the intestinal barrier by stimulating the adaptive immune response and regulates the expression of the ACE-2 enzyme. Therefore, it plays a crucial role in preventing the growth of opportunistic pathogens [45].

A low number of *Collinsella* and short-chain fatty acid-producing bacteria were associated with high mortality rates from COVID-19, and their presence was decreased in patients with severe COVID-19 compared to patients with mild COVID-19. *Collinsella* produces ursodeoxycholic acid (UDCA), which inhibits the binding of SARS-CoV-2 to the ACE-2 enzyme, suppresses pro-inflammatory cytokines, has antioxidant and antiapoptotic effects, and increases alveolar fluid clearance in acute respiratory distress syndrome. Therefore, it prevents COVID-19 infection [5,46,50].

*Blautia* increased after the resolution of the SARS-CoV-2 infection, rebalancing the composition of the gut microbiota, being positive for the patient’s recovery due to its anti-inflammatory properties [27].

**Figure 3:**
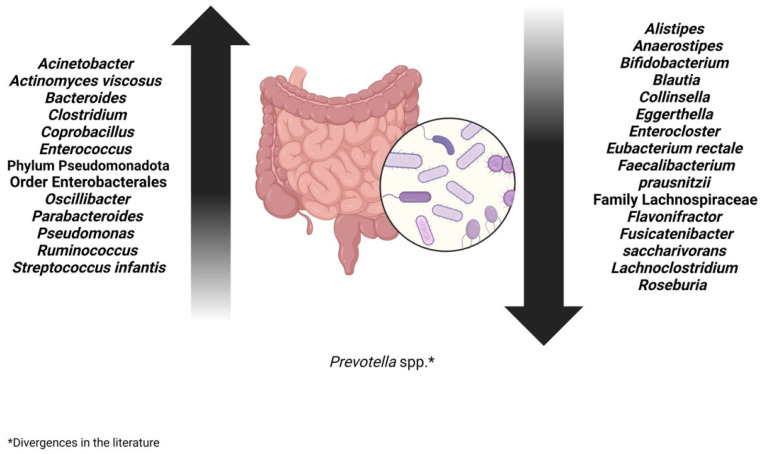
Changes in the gut microbiota in patients diagnosed with COVID-19 after the period of infection (Long-COVID).

Stool samples from patients who later recovered from COVID-19 showed an abundance of *Prevotella* spp., which is considered a biomarker in the evaluation of the host’s immune response in patients with COVID-19, contributing to a better recovery from COVID-19 [36]. However, other studies have linked an increase in *Prevotella* with a negative prognosis, as it is involved in an increase in the severity of the disease [51,52]. More in-depth studies are needed into the real function of the *Prevotella* in the context of COVID-19 (Figure 3).

## 4. Treatment Options for Intestinal Dysbiosis in Long-COVID Patients

### 4.1. Prebiotics

Normalizing intestinal dysbiosis with probiotics and prebiotics may serve as an approach to improve gastrointestinal symptoms during Long-COVID, increasing microbiome diversity and strengthening the integrity of intestinal barrier functions, consequently influencing the treatment of COVID-19 [9,35].

Prebiotics are compounds in foods that induce the growth or activity of beneficial bacteria in the gastrointestinal tract. By enriching commensal microbial populations, prebiotics contribute to improved intestinal function, immune modulation, and metabolic regulation. Although specific clinical trials investigating prebiotic use in Long-COVID are still limited, their established role in maintaining gut homeostasis supports their application as an adjunctive therapy in patients with post-COVID dysbiosis [5,9,21,37,53].

### 4.2. Probiotics

Probiotics are non-pathogenic microorganisms that, when used in appropriate doses, can improve the microecological balance, benefiting the host’s health. These benefits include inhibition of viral entry into host cells, secretion of antiviral metabolites, stimulation of innate immunity, and modulation of systemic immune responses [35,53]. In a study carried out with mice, when *Lactobacillus plantarum* was administered orally, there was protection against multiple strains of the Influenza virus, stimulating the Th1-mediated immune response, increasing the activities of Natural Killer (NK) cells, and promoting IgA-mediated mucosal immunity in the gastrointestinal and respiratory tract. Therefore, certain strains of the *Lactobacillus* can modulate immune responses to protect the host against viral respiratory infections [21].

Clinical evidence also supports the potential benefits of probiotics in COVID-19. A blinded, randomized trial comparing a four-strain probiotic formulation with maltodextrin alone reported a significant improvement in symptom remission among patients receiving probiotics, including reduced duration of fever, nausea, abdominal pain, and headaches, suggesting an influence on the gut–lung axis [36]. Wu and collaborators (2021) investigated, during the application of probiotic therapy, changes in the gut microbiota of patients with COVID-19 and pneumonia. From this, they found that daily treatment with probiotic strains of *Lactobacillus* led to a reduction in inflammatory markers such as TNF-α, IL-1β, and IL-4, and to better clinical results [54].

Oral bacteriotherapy (*Streptococcus thermophilus*, *Lactobacillus acidophilus*, *Lactobacillus helveticus*, *Lactobacillus paracasei*, *L. plantarum*, *Lactobacillus brevis*, and *Bifidobacterium lactis*—2.4 billion bacteria per day) was evaluated as a complementary therapeutic strategy to prevent the progression of COVID-19. Almost all patients who received this therapy resulted in the remission of diarrhea and other symptoms in approximately seventy-two hours, in addition to fewer patients entering the Intensive Care Unit (ICU) with the need for hospitalization or risk of mortality [55].

However, it should be noted that not all probiotics are equal and that their effectiveness in reducing mortality rates in hospitalized patients is considered undetermined. A new and more targeted approach to modulating the gut microbiota is probably needed [56]. Gutierrez-Castrellon and collaborators (2022) conducted a randomized clinical trial on patients with COVID-19 who received probiotic administration. After thirty days, total remission was observed in 53.1% of patients who received probiotics, compared to 28.1% in the placebo group. In addition to reducing nasopharyngeal viral load, probiotics also reduced the duration of digestive and non-digestive symptoms. However, no significant changes were found in the gut microbiota [57].

Despite the promising outcomes observed with the use of prebiotics and probiotics in modulating gut dysbiosis in post-COVID-19 patients, some limitations remain. In the case of probiotics, not all are likely to be the same. *Lactobacillus* and *Bifidobacterium* represent only two types of non-pathogenic bacteria, and their capacity to shift the balance of the complex gut ecosystem in combating COVID-19 remains uncertain. Therefore, a novel and more targeted approach to gut microbiota modulation may be necessary as a therapeutic strategy for COVID-19 and its comorbidities [57].

### 4.3. Symbiotic and Fecal Microbiota Transplantation (FMT)

Symbiotics, combining prebiotics and probiotics, are designed to enhance the survival and activity of beneficial microorganisms in the gastrointestinal tract. While direct evidence for symbiotic in Long-COVID remains limited, they represent a promising strategy to maximize microbiota modulation and immune support. Fecal microbiota transplantation (FMT) provides an alternative by transferring a complex microbial community from healthy donors to patients with dysbiosis, thereby restructuring the gut microbiome. FMT has been shown to increase the abundance of beneficial bacteria such as *Bifidobacterium* and *Faecalibacterium* while reducing LPS-producing bacteria, including *Intestinibacter* and members of the Prevotellaceae family [35].

A study observed around 11 patients with COVID-19 who performed FMT for four consecutive days after discharge from the hospital. Gastrointestinal symptoms decreased in 5 of 11 patients, and fecal microbiota analysis demonstrated that FMT increased the abundance of beneficial bacteria of the *Bifidobacterium* and *Faecalibacterium* [35].

Another study of 86 patients with *Clostridium difficile* infection and COVID-19 demonstrated that the combination of antibiotics and FMT provides relief from abdominal pain and decreases the levels of inflammatory cytokines compared to those treated with antibiotics alone [37]. However, there are not many published studies on FMT in the treatment of COVID-19, given the complexity of the operational process and the strict need for safety, suggesting the need for further discussions. Thus, it is necessary to establish stricter donor selection standards and apply FMT with caution, given the possible fecal-oral transmission routes [35]. Therefore, additional well-controlled clinical studies are needed to develop standardized protocols, evaluate the safety and efficacy of these therapies, and elucidate their immunological and metabolic effects in the context of post-COVID-19 patients.

Although current findings highlight the potential of microbiota-targeted therapies in Long-COVID, evidence remains limited by methodological heterogeneity, small sample sizes, and lack of standardized outcomes. Future research should prioritize large-scale randomized clinical trials, mechanistic studies on the gut–lung and gut–brain axes, and multi-omics approaches to identify specific microbial strains and metabolites involved in symptom persistence. In addition, long-term safety and efficacy of interventions such as FMT need to be carefully evaluated before broader clinical application [35].

## 5. Conclusions

The persistence of gastrointestinal symptoms in Long-COVID highlights the importance of the gut microbiota in post-infection recovery. An imbalance in the composition of microbiota can lead to an increase in opportunistic bacteria and a decrease in beneficial bacteria, which are important for maintaining and supporting the immune system. Therefore, the composition of the gut microbiota may play a role in the prognosis of patients with post-COVID-19 infection.

Therapeutic approaches such as probiotics, prebiotics, symbiotics, and FMT demonstrate potential benefits in modulating the microbiota and alleviating symptoms, although results remain heterogeneous and strain- or method-dependent. Considering the multifactorial origin of Long-COVID, further research is essential to better elucidate the mechanisms underlying gastrointestinal involvement and to develop safe and effective microbiota-targeted interventions.

The main knowledge gaps in this study include the need for longitudinal studies in humans, standardization of microbiome analysis pipelines, and results from interventional trials.

## Data Availability

The original contributions presented in this study are included in the article. Further inquiries can be directed to the corresponding author.

## References

[1] Pan American Health Organization (PAHO) (2024). Histórico da Pandemia de COVID-19. https://www.paho.org/pt/covid19/historico-da-pandemia-covid-19.

[2] World Health Organization (WHO) World Health Organization COVID-19 Deaths/WHO COVID-19 Dashboard. https://data.who.int/dashboards/covid19/deaths?n=o.

[3] Moreno-Corona N.C., López-Ortega O., Pérez-Martínez C.A., Martínez-Castillo M., De Jesús-González L.A., León-Reyes G., León-Juárez M. (2023). Dynamics of the Microbiota and Its Relationship with Post-COVID-19 Syndrome. Int. J. Mol. Sci..

[4] De R., Dutta S. (2022). Role of the Microbiome in the Pathogenesis of COVID-19. Front. Cell. Infect. Microbiol..

[5] Gang J., Wang H., Xue X., Zhang S. (2022). Microbiota and COVID-19: Long-Term and Complex Influencing Factors. Front. Microbiol..

[6] Ailioaie L.M., Ailioaie C., Litscher G. (2023). Gut Microbiota and Mitochondria: Health and Pathophysiological Aspects of Long COVID. Int. J. Mol. Sci..

[7] Ashktorab H., Challa S.R., Singh G., Nanduri S., Ibrahim M., Martirosyan Z., Whitsell P., Chirumamilla L.G., Shayegh N., Watson K. (2023). Gastrointestinal Manifestations and Their Association with Neurologic and Sleep Problems in Long COVID-19 Minority Patients: A Prospective Follow-Up Study. Dig. Dis. Sci..

[8] Giovanetti M., Pannella G., Altomare A., Rocchi G., Guarino M., Ciccozzi M., Riva E., Gherardi G. (2024). Exploring the Interplay Between COVID-19 and Gut Health: The Potential Role of Prebiotics and Probiotics in Immune Support. Viruses.

[9] Zuo T., Wu X., Wen W., Lan P. (2021). Gut Microbiome Alterations in COVID-19. Genom. Proteom. Bioinform..

[10] Suskun C., Kilic O., Yilmaz Ciftdogan D., Guven S., Karbuz A., Ozkaya Parlakay A., Kara Y., Kacmaz E., Sahin A., Boga A. (2022). Intestinal Microbiota Composition of Children with Infection with Severe Acute Respiratory Syndrome Coronavirus 2 (SARS-CoV-2) and Multisystem Inflammatory Syndrome (MIS-C). Eur. J. Pediatr..

[11] Hastie C.E., Lowe D.J., McAuley A., Mills N.L., Winter A.J., Black C., Scott J.T., O’Donnell C.A., Blane D.N., Browne S. (2023). True prevalence of long-COVID in a nationwide, population cohort study. Nat. Commun..

[12] Yeoh Y.K., Zuo T., Lui G.C.-Y., Zhang F., Liu Q., Li A.Y., Chung A.C., Cheung C.P., Tso E.Y., Fung K.S. (2021). Gut Microbiota Composition Reflects Disease Severity and Dysfunctional Immune Responses in Patients with COVID-19. Gut.

[13] Checa-Ros A., Jeréz-Calero A., Molina-Carballo A., Campoy C., Muñoz-Hoyos A. (2021). Current Evidence on the Role of the Gut Microbiome in ADHD Pathophysiology and Therapeutic Implications. Nutrients.

[14] Chatterjee P., Garcia M.A., Cote J.A., Yun K., Legerme G.P., Habib R., Tripepi M., Young C., Kulp D., Dyall-Smith M. (2024). Involvement of ArlI, ArlJ, and CirA in Archaeal Type IV Pilin-Mediated Motility Regulation. J. Bacteriol..

[15] Donati Zeppa S., Agostini D., Piccoli G., Stocchi V., Sestili P. (2020). Gut Microbiota Status in COVID-19: An Unrecognized Player?. Front. Cell. Infect. Microbiol..

[16] Ferreira-Junior A.S., Borgonovi T.F., Salis L.V.V., Leite A.Z., Dantas A.S., De Salis G.V.V., Cruz G.N.F., De Oliveira L.F.V., Gomes E., Penna A.L.B. (2022). Detection of Intestinal Dysbiosis in Post-COVID-19 Patients One to Eight Months after Acute Disease Resolution. Int. J. Environ. Res. Public Health.

[17] Rueda Ruzafa L., Cedillo J.L., Hone A.J. (2021). Nicotinic Acetylcholine Receptor Involvement in Inflammatory Bowel Disease and Interactions with Gut Microbiota. Int. J. Environ. Res. Public Health.

[18] Verma A., Bhagchandani T., Rai A., Nikita, Sardarni U.K., Bhavesh N.S., Gulati S., Malik R., Tandon R. (2024). Short-Chain Fatty Acid (SCFA) as a Connecting Link Between Microbiota and Gut-Lung Axis—A Potential Therapeutic Intervention to Improve Lung Health. ACS Omega.

[19] Zhang F., Wan Y., Zuo T., Yeoh Y.K., Liu Q., Zhang L., Zhan H., Lu W., Xu W., Lui G.C.Y. (2022). Prolonged Impairment of Short-Chain Fatty Acid and L-Isoleucine Biosynthesis in Gut Microbiome in Patients with COVID-19. Gastroenterology.

[20] Włodarczyk J., Czerwiński B., Fichna J. (2022). Short-chain fatty acids-microbiota crosstalk in the coronavirus disease (COVID-19). Pharmacol. Rep..

[21] Ngo V.L., Gewirtz A.T. (2021). Microbiota as a Potentially-Modifiable Factor Influencing COVID-19. Curr. Opin. Virol..

[22] Karlsson A.C., Humbert M., Buggert M. (2020). The known unknowns of T cell immunity to COVID-19. Sci. Immunol..

[23] Fajgenbaum D.C., June C.H. (2020). Cytokine Storm. N. Engl. J. Med..

[24] Yamamoto S., Saito M., Tamura A., Prawisuda D., Mizutani T., Yotsuyanagi H. (2021). The Human Microbiome and COVID-19: A Systematic Review. PLoS ONE.

[25] Zrelli S., Amairia S., Zrelli M. (2021). Respiratory syndrome coronavirus-2 response: Microbiota as *Lactobacilli* could make the difference. J. Med. Virol..

[26] Ma P.J., Wang M.M., Wang Y. (2022). Gut microbiota: A new insight into lung diseases. Biomed. Pharmacother..

[27] De Maio F., Ianiro G., Coppola G., Santopaolo F., Abbate V., Bianco D.M., Del Zompo F., De Matteis G., Leo M., Nesci A. (2021). Improved Gut Microbiota Features After the Resolution of SARS-CoV-2 Infection. Gut Pathog..

[28] Ancona G., Alagna L., Alteri C., Palomba E., Tonizzo A., Pastena A., Muscatello A., Gori A., Bandera A. (2023). Gut and Airway Microbiota Dysbiosis and Their Role in COVID-19 and Long-COVID. Front. Immunol..

[29] Kaushal A., Noor R. (2022). Association of Gut Microbiota with Inflammatory Bowel Disease and COVID-19 Severity: A Possible Outcome of the Altered Immune Response. Curr. Microbiol..

[30] Meringer H., Wang A., Mehandru S. (2023). The Pathogenesis of Gastrointestinal, Hepatic, and Pancreatic Injury in Acute and Long Coronavirus Disease 2019 Infection. Gastroenterol. Clin. N. Am..

[31] Mehandru S., Merad M. (2022). Pathological Sequelae of Long-Haul COVID. Nat. Immunol..

[32] Lau H.C.-H., Ng S.C., Yu J. (2022). Targeting the Gut Microbiota in Coronavirus Disease 2019: Hype or Hope?. Gastroenterology.

[33] Vignesh R., Swathirajan C.R., Tun Z.H., Rameshkumar M.R., Solomon S.S., Balakrishnan P. (2021). Could Perturbation of Gut Microbiota Possibly Exacerbate the Severity of COVID-19 via Cytokine Storm?. Front. Immunol..

[34] Ivanov I.I., Tuganbaev T., Skelly A.N., Honda K. (2022). T Cell Responses to the Microbiota. Annu. Rev. Immunol..

[35] Zhou B., Pang X., Wu J., Liu T., Wang B., Cao H. (2023). Gut Microbiota in COVID-19: New Insights from Inside. Gut Microbes.

[36] Wais T., Hasan M., Rai V., Agrawal D.K. (2022). Gut-Brain Communication in COVID-19: Molecular Mechanisms, Mediators, Biomarkers, and Therapeutics. Expert Rev. Clin. Immunol..

[37] He K.-Y., Lei X.-Y., Zhang L., Wu D.-H., Li J., Lü L.Y., Laila U.E., Cui C.-Y., Xu Z., Jian Y.P. (2023). Development and Management of Gastrointestinal Symptoms in Long-Term COVID-19. Front. Microbiol..

[38] Hamrefors V., Kahn F., Holmqvist M., Carlson K., Varjus R., Gudjonsson A., Fedorowski A., Ohlsson B. (2024). Gut Microbiota Composition Is Altered in Postural Orthostatic Tachycardia Syndrome and Post-Acute COVID-19 Syndrome. Sci. Rep..

[39] Nayak B.N., Singh R.B., Buttar H.S., Singh R.B., Watanabe S., Isaza A.A. (2022). Chapter 51—Biochemical and Dietary Functions of Tryptophan and Its Metabolites in Human Health. Functional Foods and Nutraceuticals in Metabolic and Non-Communicable Diseases.

[40] Blackett J.W., Sun Y., Purpura L., Margolis K.G., Elkind M.S.V., O’Byrne S., Wainberg M., Abrams J.A., Wang H.H., Chang L. (2022). Decreased Gut Microbiome Tryptophan Metabolism and Serotonergic Signaling in Patients with Persistent Mental Health and Gastrointestinal Symptoms After COVID-19. Clin. Transl. Gastroenterol..

[41] Michaelis S., Zelzer S., Schnedl W.J., Baranyi A., Meinitzer A., Enko D. (2022). Assessment of tryptophan and kynurenine as prognostic markers in patients with SARS-CoV-2. Clin. Chim. Acta.

[42] Li J., Jing Q., Li J., Hua M., Di L., Song C., Huang Y., Wang J., Chen C., Wu A.R. (2023). Assessment of Microbiota in the Gut and Upper Respiratory Tract Associated with SARS-CoV-2 Infection. Microbiome.

[43] Zhou B., Yuan Y., Zhang S., Guo C., Li X., Li G., Xiong W., Zeng Z. (2020). Intestinal Flora and Disease Mutually Shape the Regional Immune System in the Intestinal Tract. Front. Immunol..

[44] Ghoshal U.C., Ghoshal U. (2023). Gastrointestinal Involvement in Post-Acute Coronavirus Disease (COVID)-19 Syndrome. Curr. Opin. Infect. Dis..

[45] Brīvība M., Silamiķele L., Birzniece L., Ansone L., Megnis K., Silamiķelis I., Pelcmane L., Borisova D., Rozenberga M., Jagare L. (2024). Gut Microbiome Composition and Dynamics in Hospitalized COVID-19 Patients and Patients with Post-Acute COVID-19 Syndrome. Int. J. Mol. Sci..

[46] Liu Q., Mak J.W.Y., Su Q., Yeoh Y.K., Lui G.C.-Y., Ng S.S.S., Zhang F., Li A.Y.L., Lu W., Hui D.S.-C. (2022). Gut Microbiota Dynamics in a Prospective Cohort of Patients with Post-Acute COVID-19 Syndrome. Gut.

[47] Zuo T., Liu Q., Zhang F., Lui G.C.-Y., Tso E.Y., Yeoh Y.K., Chen Z., Boon S.S., Chan F.K., Chan P.K. (2020). Depicting SARS-CoV-2 Faecal Viral Activity in Association with Gut Microbiota Composition in Patients with COVID-19. Gut.

[48] Ailioaie L.M., Ailioaie C., Litscher G. (2023). Infection, Dysbiosis and Inflammation Interplay in the COVID Era in Children. Int. J. Mol. Sci..

[49] Nguyen L.H., Okin D., Drew D.A., Battista V.M., Jesudasen S.J., Kuntz T.M., Bhosle A., Thompson K.N., Reinicke T., Lo C.-H. (2023). Metagenomic Assessment of Gut Microbial Communities and Risk of Severe COVID-19. Genome Med..

[50] Hirayama M., Nishiwaki H., Hamaguchi T., Ito M., Ueyama J., Maeda T., Kashihara K., Tsuboi Y., Ohno K. (2021). Intestinal *Collinsella* May Mitigate Infection and Exacerbation of COVID-19 by Producing Ursodeoxycholate. PLoS ONE.

[51] Zhang D., Weng S., Xia C., Ren Y., Han X., Xu Y., Yang X., Wu R., Peng L., Sun L. (2023). Gastrointestinal Symptoms of Long COVID-19 Related to the Ectopic Colonization of Specific Bacteria That Move Between the Upper and Lower Alimentary Tract and Alterations in Serum Metabolites. BMC Med..

[52] Khan A.A., Khan Z. (2020). COVID-2019-Associated Overexpressed *Prevotella* Proteins Mediated Host–Pathogen Interactions and Their Role in Coronavirus Outbreak. Bioinformatics.

[53] Alharbi K.S., Singh Y., Hassan Almalki W., Rawat S., Afzal O., Alfawaz Altamimi A.S., Kazmi I., Al-Abbasi F.A., Alzarea S.I., Singh S.K. (2022). Gut Microbiota Disruption in COVID-19 or Post-COVID Illness Association with Severity Biomarkers: A Possible Role of Pre/Pro-Biotics in Manipulating Microflora. Chem. Biol. Interact..

[54] Wu C., Xu Q., Cao Z., Pan D., Zhu Y., Wang S., Liu D., Song Z., Wei J., Ruan Y. (2021). The Volatile and Heterogeneous Gut Microbiota Shifts of COVID-19 Patients over the Course of a Probiotics-Assisted Therapy. Clin. Transl. Med..

[55] d’Ettorre G., Ceccarelli G., Marazzato M., Campagna G., Pinacchio C., Alessandri F., Ruberto F., Rossi G., Celani L., Scagnolari C. (2020). Challenges in the Management of SARS-CoV2 Infection: The Role of Oral Bacteriotherapy as Complementary Therapeutic Strategy to Avoid the Progression of COVID-19. Front. Med..

[56] Mak J.W.Y., Chan F.K.L., Ng S.C. (2020). Probiotics and COVID-19: One Size Does Not Fit All. Lancet Gastroenterol. Hepatol..

[57] Gutiérrez-Castrellón P., Gandara-Martí T., Abreu Y Abreu A.T., Nieto-Rufino C.D., López-Orduña E., Jiménez-Escobar I., Jiménez-Gutiérrez C., López-Velazquez G., Espadaler-Mazo J. (2022). Probiotic Improves Symptomatic and Viral Clearance in COVID-19 Outpatients: A Randomized, Quadruple-Blinded, Placebo-Controlled Trial. Gut Microbes.

